# Proposal for a preventive protocol for medication-related osteonecrosis of the jaw

**DOI:** 10.4317/medoral.24197

**Published:** 2020-10-09

**Authors:** Manuel Mª Romero-Ruiz, Marta Romero-Serrano, Ascensión Serrano-González, María Ángeles Serrera-Figallo, José Luis Gutiérrez-Pérez, Daniel Torres-Lagares

**Affiliations:** 1 DDS. Dental School. University of Seville, Spain; 2 MD, PhD. Hospital Puerta del Mar, Cádiz, Spain; 3 DDS. PhD. Dental School. University of Seville, Spain; 4 DMD, PhD. Dental School. University of Seville, Spain

## Abstract

**Background:**

Medication-related osteonecrosis of the jaw (MRONJ) is a severe adverse reaction experienced by some patients exposed to certain drugs (antiresorptives such as bisphosphonates or denosumab, and antiangiogenic drugs). From a review of the literature it appears that there is no uniform criterion when selecting preventive measures; these vary according to author. Likewise, the measures recommended are usually general, so that in few cases they result in specific actions to be applied depending on the different variables involved such as the type of drug used, the duration of its application, the underlying pathology, the presence or absence of risk factors, etc. The aim of this study has been to design a preventive protocol which can be easily applied in any clinic or by any dental care service.

**Material and Methods:**

We undertook an exhaustive literature review to find any articles related to the topic of study, namely, preventive measures for medication-related osteonecrosis of the jaw, on the one hand generically and on the other focusing on dental implant treatment. The most part the criteria of the Preferred Reporting Items for Systematic Reviews and Meta-Analyses (PRISMA) guidelines were followed. From 3946 items, we selected a total of 21 items.

**Results:**

From the analysis of the selected articles, several protocols have been developed that are easy to apply in a dental clinic.: Protocol 1. Before starting treatment with antiresorptives (Patients who are going to be treated for osteoporosis / Patients who are going to be treated for cancer). Protocol 2. Once treatment is initiated with antiresorptives (Patients being treated for osteoporosis / Patients being treated for cancer).

**Conclusions:**

The application of these protocols requires an interdisciplinary team which can handle the various treatments and apply the measures contained in them. Along with a team of well-educated and trained dentists, it is equally important to maintain contact with the medical team involved in the treatment of the underlying pathology, especially rheumatologists, oncologists, internists and gynaecologists. All the above requires a great staff learning and organization effort, continuous training and coordination of the whole team involved in the preventive management of these patients.

** Key words:**Medication-related osteonecrosis of the jaw, clinical protocols, clinical guidelines, prevention.

## Introduction

Medication-related osteonecrosis of the jaw (MRONJ) is a severe adverse reaction experienced by some patients exposed to certain drugs (antiresorptives such as bisphosphonates or denosumab, and antiangiogenic drugs), used in cases of osteoporosis or bone manifestations in different types of cancer, to reduce skeletal complications of these conditions, achieving a reduction in pain and typical pathological fractures, as well as an improvement in the life quality of these patients (1).

According to the American Association of Oral and Maxillofacial Surgeons (AAOMS 2014), patients with MRONJ should be or have been in treatment with antiresorptive or antiangiogenic drugs, present exposed bone or bone which may be probed through an intra- or extraoral fistula in the maxillofacial region, and the lesion must have persisted for more than 8 weeks with no history of radiotherapy in the region (2).

The etiopathogenesis of this type of Osteonecrosis of the Jaw (ONJ) nowadays continues to be a challenge for researchers, being a constant topic of debate. From the data available it may be deduced that the aetiology would be multifactorial, there being on the one hand inhibition of the osteoclast function by the antiresorptive drugs, which would lead to disorders in the repairing, healing and bone remodelling mechanisms, essential in protecting against infection, and microfractures which take place as a result of physiological bone function (3). On the other hand, both the antiangiogenic drugs such as Bevacizumab or Sunitinib, and some bisphosphonates such as zoledronic acid are capable of inhibiting angiogenesis, by reducing the formation of blood vessels, which is fundamental for healing and bone remodelling (4).

In recent years, the infectious/inflammatory theory has become increasingly important as a cause for the emergence of ONJ. Different studies on animal models support the theory that infection or local inflammation could trigger a condition of osteonecrosis in these patients (3). Although it is well known that the majority of cases of ONJ had a dental extraction history, it is also true that normally these extracted teeth had undergone prior periodontal or periapical pathological infection, which justified their extraction. Given that most teeth with a dental inflammatory disease are eventually extracted, there may be confusion on the true role of the surgical procedure itself as a direct trigger for ONJ (2,5,6). The basic role of infection in the pathogenesis of this condition is manifested by the fact that its incidence is reduced as soon as the dental hygiene of these patients improves (7). The mechanism by which microorganisms induce ONJ could be related to the production by the bacteria in certain substances such as lipopolysaccharides which would favour reabsorption, or Receptor Activator of Nuclear Factor Kappa B Ligand (RANKL) in fibroblasts, having the same effect. Similarly, local acidosis induced by infection has also been related as a cause of the release of bisphosphonates, facilitating osteonecrosis (8,9).

Treatment with dental implants in patients who take antiresorptives or antiangiogenic drugs has always been a controversial topic. As cases of MRONJ were being published, it was highlighted that in an elevated percentage, the precipitating factor was a dental extraction (54%-61%), such that although there was not much evidence, it was deemed that the risk of triggering an ONJ after dentoalveolar surgery would be similar to the one that existed after exodontia. In this sense, the surgical procedure of inserting an implant in these patients would involve a risk of ONJ similar to that of dental exodontia (2,10). Slowly publications began to appear which related implants with the emergence of ONJ, arousing controversy about the desirability of recommending implantological treatments in patients treated with antiresorptives, although the evidence in that respect is heterogeneous, incomplete and of low quality (11,12). There is sufficient evidence to state that the risk of implant failure due to ONJ is limited in patients undergoing treatment with antiresorptives for osteoporosis, although the risk must be assessed on an individual basis. However, in patients undergoing treatment with antiresorptives for cancer, the risk is much higher and there is a consensus that implants should be contraindicated in these cases (2,13,14).

Notwithstanding, from the evidence published in recent years, it appears that the majority of cases of peri-implant MRONJ develop as a late complication around previously osseointegrated and successfully loaded implants, such that the condition could not be attributed to the surgical procedure of implant insertion. Several publications have suggested that the presence of peri-implantitis could be a more important risk factor for MRONJ than surgical insertion, which would reinforce the importance of the infectious/inflammatory theory in the etiopathogenesis of MRONJ in these cases (14-18).

Treatment of MRONJ once established is complex, because it depends on the stage of the disease, there being several therapeutic approaches, sometimes conflicting, depending on the authors undertaking it. Bermúdez *et al*. (19) carried out a study on the different therapeutic approaches found in the literature and grouped them into seven protocols, each one of which covered different types of treatment, highlighting that the best results were obtained with a conservative protocol, with clinical and radiological follow-up, minimally invasive surgical treatment and various coadjuvant measures. This shows the enormous variety of existing proposals and the difficulty in tackling the process therapeutically. In part due to the above, when talking of therapeutic management of these patients, stress has been laid on the importance of a multi-disciplinary approach which should include consulting qualified dental professionals, when deciding on treating a patient with antiresorptives or antiangiogenics. There is increasing evidence that early screening, the application of adequate preventive measures and correct dental care before initiating antiresorptive treatment, achieve a reduction in the incidence of MRONJ using guidelines covering educational aspects and ones aimed at motivating patients to take part in their dental healthcare, as well as measures targeted at eliminating or preventing infected dental, periodontal and peri-implant sites (2,10,20-22). Likewise, preventive protocols for performing surgical extractions with the least possible trauma have been described, using antibiotic prophylaxis, finding a reduction in the risk of osteonecrosis (23,24).

However, from a review of the literature it appears that there is no uniform criterion when selecting preventive measures; these vary according to author. Likewise, the measures recommended are usually general, so that in few cases they result in specific actions to be applied depending on the different variables involved such as the type of drug used, the duration of its application, the underlying pathology, the presence or absence of risk factors, etc. A similar situation arises with follow-up times, when check-ups should be carried out, or with the drugs and preventive measures employed before an exodontia or any other surgical procedure in these patients.

We have not found in the literature any clearly defined, wide-ranging protocol which outlines specifically and systematically the different preventive measures for MRONJ set out in published studies in the literature, and especially for patients who are carriers or who wish to receive treatment with dental implants. Therefore, the aim of this study has been to design a preventive protocol which can be easily applied in any clinic or by any dental care service; one which is systematic and detailed and which takes into consideration all the variables involved in those patients who have received or are receiving treatment with antiresorptive or antiangiogenic drugs, and who are wearers or are about to receive treatment with dental implants.

## Material and Methods

- Protocols and eligibility criteria

We undertook an exhaustive literature review to find any articles related to the topic of study, namely, preventive measures for medication-related osteonecrosis of the jaw, on the one hand generically and on the other focusing on dental implant treatment. Although for the most part the criteria of the Preferred Reporting Items for Systematic Reviews and Meta-Analyses (PRISMA) guidelines were followed, this review cannot be considered strictly systematic, due to the large number of variables involved in the search, given that our aim was to draw up a preventive protocol describing all the measures published in the literature. As a result of the huge spread of the data in the published articles and the heterogeneous nature of these, we deemed it inappropriate to ask a specific PICO question because we ran the risk of leaving out articles relevant to our search. For this reason, likewise, we had to resort to review or expert opinion articles, which placed more emphasis on the specific preventive measures we wished to include in the protocol.

The inclusion criteria were: (a) Studies published between January 2003 and 30 January 2019; (b) Human studies; (c) Any language; (d) case series, cohort studies, case-control studies, and controlled and/or randomized controlled clinical trials (CTs/RCTs); (e) retro or prospective studies; (f) studies including patients having undergone or undergoing oral or parenteral antiresorptive or antiangiogenic drugs, with or without implant treatment, to whom any type of protocol or preventive measure was being applied; (g) review articles, systematic reviews and meta-analysis on the application of preventive measures or protocols for MRONJ in patients having taken, taking or planning to take the drugs involved. The following exclusion criteria were applied: (a) that they did not meet the inclusion criteria; (b) animal studies; (c) case reports.

- Search sources and strategy

An electronic search was conducted using three databases, PubMed, (Medline), Embase (Ovid) and Cochrane database of systematic reviews. The review was completed with a manual search in scientific journals in this sector in the e-library of the University of Seville. Likewise lists of references in all the publications identified were reviewed.

Search of the Medline (PubMed) database was carried out using MeSH (Medical Subjects Headings) terms and free terms, in different combinations using Boolean Operators “AND” and “OR”. The terms used were general terms; (“Dental” OR “Oral”). Terms related to drugs involved; (“Diphosphonates” OR “Bisphosphonates” OR “Alendronic Acid” OR “Alendronate” OR “Etidronic Acid” OR “Etidronate” OR “Ibandronic Acid” OR “Ibandronate” OR “Pamidronate” OR “Risedronic Acid” OR “Risedronate” OR “Zoledronic Acid” OR “Zoledronate” OR “Denosumab” OR “Human monoclonal antibody to RANKL” OR “RANK ligand” OR “RANK antibody” OR “Bevacizumab” or “Sunitinib” OR “Antiresorptive drugs” OR “Antiresorptive agents” OR “Angiogenesis inhibitor”. Terms related to Osteonecrosis of the jaw; “Bisphosphonate-associated osteonecrosis of the jaw” OR “Medication related osteonecrosis” OR “Jaw osteonecrosis” OR “Osteonecrosis” OR “MRONJ” OR” BRONJ”. Terms related to dental implants; “Dental implants” OR “Dental implant” OR “Dental implants adverse effects” OR “Implant treatment” OR “Implant therapy” OR “Implants” OR “Osseointegration” OR “Osseointegrated dental implantation” OR “Dental implantation, endosseus” OR “Implant loss” OR “Implant failure” OR “Periimplantitis” OR “Peri-implantitis” OR “Periimplant disease” OR “. Terms related to dental extraction or oral surgery as a risk factor; “Tooth extraction” OR “Tooth extractions” OR “Dental extraction” OR “Oral surgery” OR “Oral surgery procedure” OR “Oral surgery procedures” OR “Procedures, oral surgery”. Terms related to prevention or preventive measures for osteonecrosis; “Preventive dentistry” OR “Prophylaxis” OR “Dental Prophylaxis” OR “Prophylaxis, dental” OR “Preventive measures” OR “Preventive management” OR “Antibiotic Prophylaxis” OR “Antibiotic” OR “Bisphosphonates-associated osteonecrosis of the jaw therapy” OR “Bisphosphonates-associated osteonecrosis of the jaw prevention and control” OR “Bisphosphonates-associated osteonecrosis of the jaw preventive protocol” OR ”Preventive protocol” OR “Preventive” OR “Protocol”. For the other two databases, similar terms were used but adapted to the specific criteria of each of them.

- Data gathering and extraction

Two authors (MMRR and MRS) reviewed all the titles and abstracts independently. After ruling out all those which did not meet the eligibility criteria, the complete text of the remaining articles was reviewed. The complete text of those which offered little information in the title or abstract were also selected, to avoid missing out any relevant article. Any disagreements were resolved by discussion between the two reviewers.

## Results

- Articles selected

**Figure 1:**
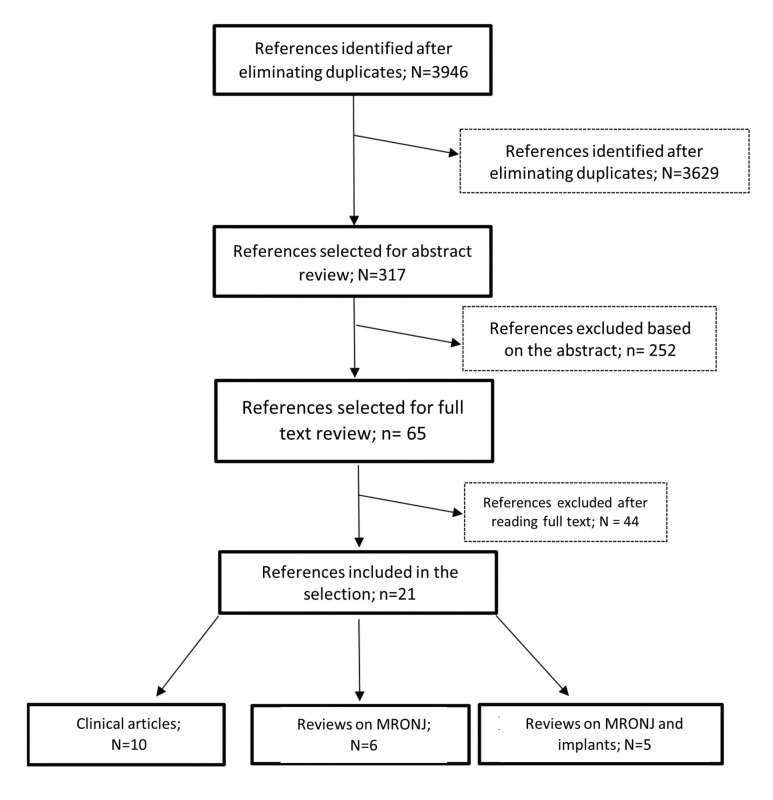
Flowchart of the search and inclusion process for studies for review.

Fig. 1 shows the flowchart of the search process. Of the 3946 initial articles, after the various exclusion processes, 21 articles were selected which met the inclusion criteria. The articles included were grouped into: clinical articles (n=10, Table 1); (Dimopoulos *et al*., 2009 (20); Ripamonti *et al*., 2009 (25); Lodi *et al*., 2010 (26); Ferlito *et al*., 2011 (27); Kwon *et al*., 2012 (28); Vandone *et al*., 2012 (29); Bramati *et al*., 2014 (30); Troeltzsch *et al*., 2016 (31); Giovannacci *et al*., 2016 (13); Mücke *et al*., 2016 (32)). Review articles on MRONJ (n=6, Table 2); (Ruggiero *et al*., 2014 (2); Otto *et al*., 2015 (33); Diniz- Freitas *et al*. 2016 (34); Beth-Tasgodan *et al*., 2017 (35); Di Fede *et al*., 2018 (36); Karna *et al*., 2018 (37)). Review articles on relation between MRONJ and dental implants (n=5, Table 3); (Ata-Ali *et al*., 2016 (11); Freitas *et al*., 2016 (38); Walter *et al*., 2016 (18); Guazzo *et al*., 2017 (39); Stavropoulos *et al*., 2018 (1)).

**Table 1:**
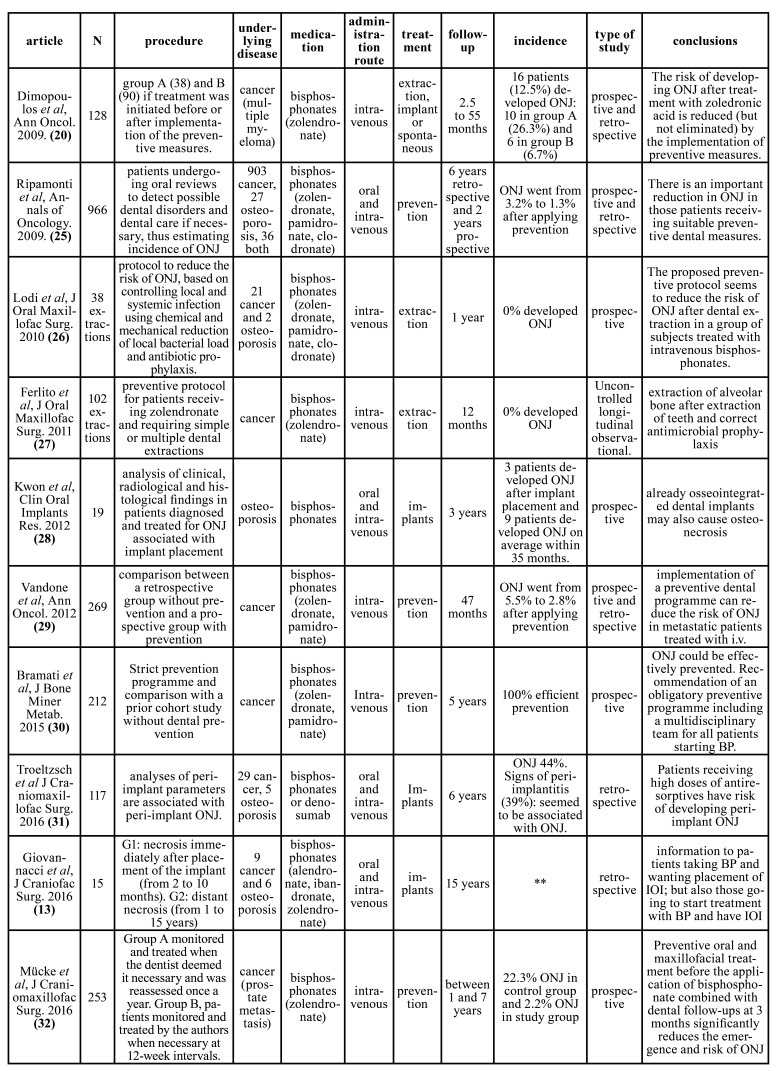
Clinical articles.

**Table 2:**
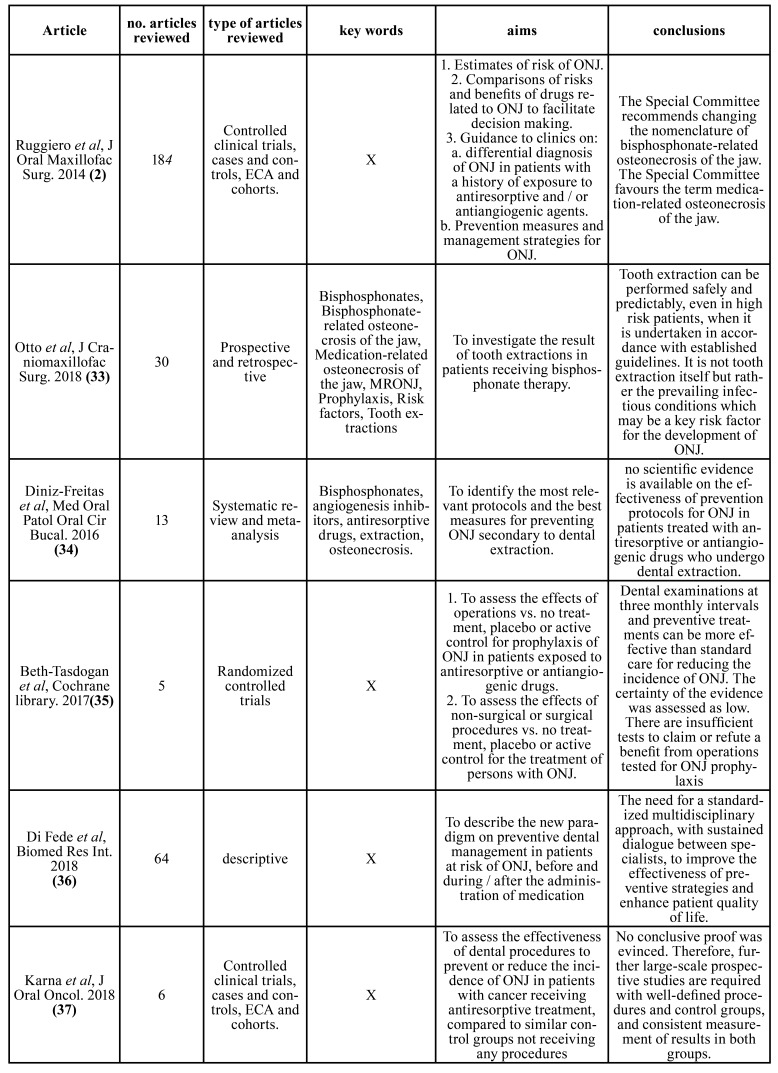
Review articles on osteonecrosis.

**Table 3:**
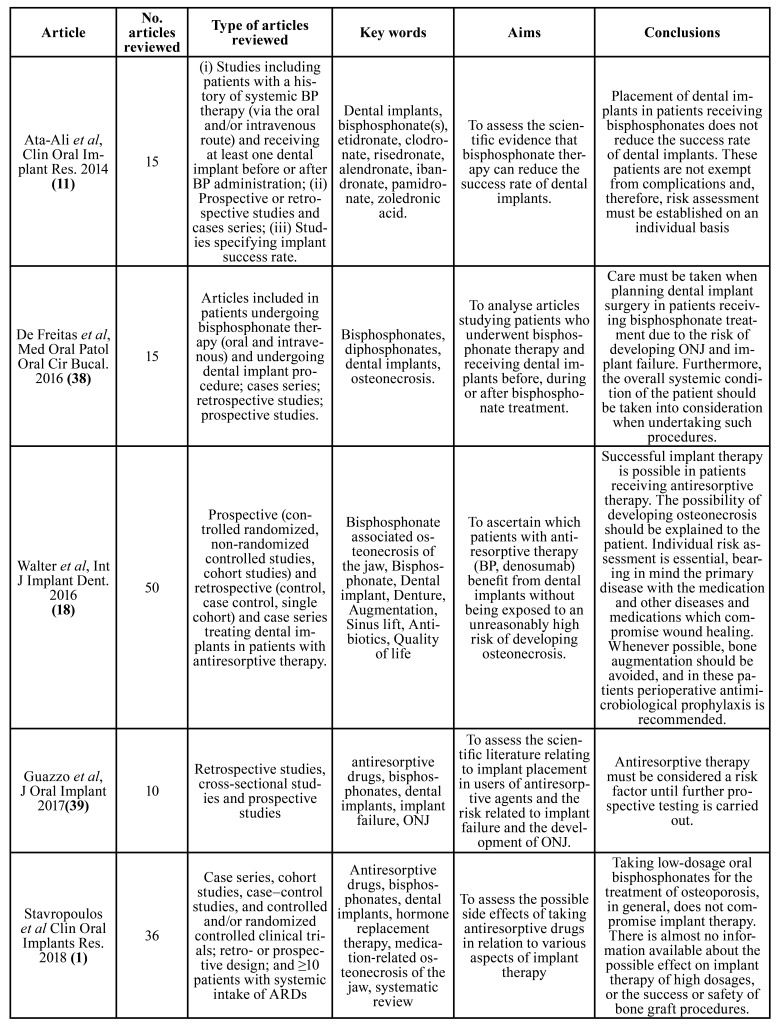
Review articles on osteonecrosis and implants.

- Preventive protocols

The different variables involved were grouped into two preventive protocols; one for patients who had not yet begun treatment with antiresorptive drugs and the other for those who were already being treated with said drugs. Each group comprised in turn two subgroups depending on whether they were patients treated for osteoporosis or for cancer.

Protocols, for patients already treated or who wished to be treated with dental implants, are outlined below.

**Figure 2:**
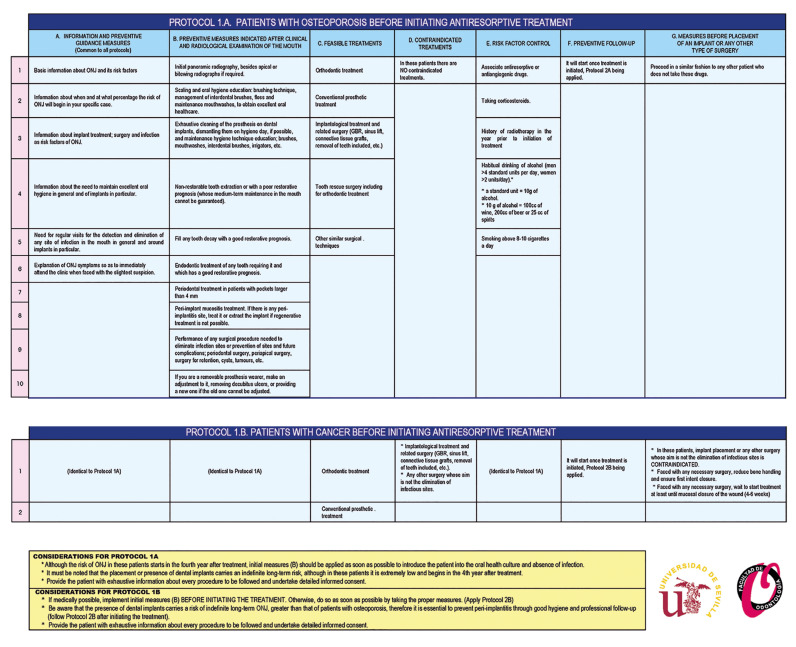
Protocols 1A and 1B.

PROTOCOL 1. BEFORE STARTING TREATMENT WITH ANTIRESORPTIVES (Fig. 2)

A. PATIENTS WHO ARE GOING TO BE TREATED FOR OSTEOPOROSIS.

B. PATIENTS WHO ARE GOING TO BE TREATED FOR CANCER.

**Figure 3:**
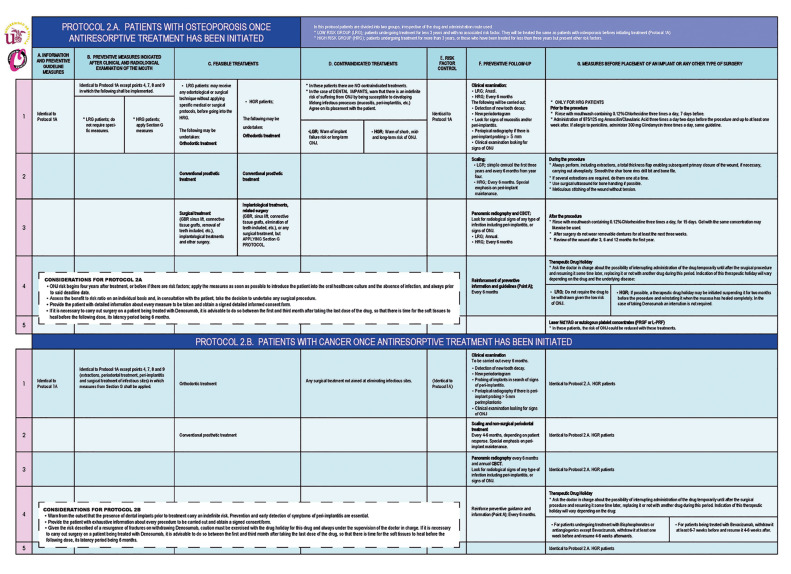
Protocols 2A and 2B.

PROTOCOL 2. ONCE TREATMENT IS INITIATED WITH ANTIRESORPTIVES (Fig. 3)

A. PATIENTS BEING TREATED FOR OSTEOPOROSIS

B. PATIENTS BEING TREATED FOR CANCER

## Discussion

Antiresorptive drugs have begun to cover an important therapeutic field in two broad groups of patients, those affected by osteoporosis from various sources and those who suffer from oncological osteolytic processes. These conditions have in common the loss of bone density and the possibility of pathological fractures emerging which considerably compromise quality of life and entail high morbidity and elevated therapeutic costs, amongst other problems. These drugs have demonstrated their capacity for reducing bone symptoms although in certain cases they can induce osteonecrotic lesions of the jaw as an undesired effect of their use, possibly leading to serious consequences for the patient (2).

Even though the risk of suffering an ONJ in patients with osteoporosis is very low (between 0.1 and 0.21 according to different series), in recent years alerts have been raised about how misleading this data is, since the number of persons undergoing treatment for osteoporosis is very elevated, it is a chronic treatment, and the risk of ONJ increases over the time the drug is taken, these being reasons why some authors point to the frequency of ONJ in these patients being greater than initially suspected (33). Warnings have been issued about the fact that many patients treated with antiresorptives for osteoporosis, do not meet the criteria established for prescribing said medication, which is why the prevention of ONJ should start by unifying criteria across different medical professionals for proper prescription of the drugs which produce it and thus avoid cases of unnecessary treatment. Otherwise, the risk of ONJ in patients with certain cancers is much greater (0.7% - 7.7 % according to series) so although it is advisable to apply preventive measures in all cases, in these patients it is important to maximize them.

Etiopathogenetic mechanisms are still controversial, different etiopathogenetic theories having been postulated to explain the emergence of ONJ (9,34). It is clear that dentoalveolar surgery involves an aggression to a bone depleted of its remodelling functions by the lack of osteoclasts, which would prevent it from coping with demands, which together with antiangiogenesis caused by drugs could justify osteonecrosis. However, data exist that contradict this theory such as the fact that the significant reduction in osteoclastic activity mediated by these drugs would induce a predominance of osteoblastic activity and therefore would lead more towards osteopetrosis than towards osteonecrosis. Likewise, in conditions such as hyperparathyroidism in which bone turnover is also reduced, osteonecrosis does not occur, however there are patients described with ONJ in which said turnover is normal (7,31). In this context, ever more data reinforce the role of infectious-inflammatory processes in the development of osteonecrosis, which is manifested in the significant decrease in the incidence of ONJ reported by many authors in patients to whom preventive measures are applied aimed at improving oral hygiene and reducing infectious processes in these patients (20,25-30). These data justify of themselves the need for applying preventive measures for infectious-inflammatory conditions in these patients as part of their therapeutic management and manifest the importance of having systematic protocols which can be routinely applied to these patients.

It has been suggested that microorganisms could induce bone resorption in ONJ by producing certain substances such as lipopolysaccharides which favour resorption, or receptor activators of nuclear factor-κB ligand (RANKL) in fibroblasts, having the same effect (8,15). Likewise, local acidosis induced by infection has been indicated as the cause of the release of bisphosphonates on bone, facilitating osteonecrosis (9). Macrophages and monocytes could intervene in the necrosis mechanism such that by culturing them with solutions of bisphosphonates it has been postulated that these would phagocyte before the macrophages, which would lose their function of responding to the infection (40). All this has led to taking extreme measures against infectious processes to try to reduce the incidence of ONJ in these patients, promoting the application of preventive measures to facilitate the elimination of said sites or their prevention by establishing proper oral healthcare.

Placement of a dental implant and surgery associated with this type of treatment is deemed, from the outset, a risk for the emergence of ONJ in susceptible patients, the same as any other surgical procedure, several cases of ONJ having been published after the placement of implants in recent years (16,28). This gave rise to controversy over whether it was appropriate or not to recommend this type of treatment in patients undergoing antiresorptive therapy. Nowadays there is sufficient evidence to affirm that the risk of implant failure caused by osteonecrosis is limited in patients with osteoporosis undergoing treatment with low doses of antiresorptives (1,11,15,18). However, although data is lacking, the risk for patients taking antiresorptives for cancerous lesions -much higher doses-, is considerably more elevated, consequently there is a consensus for stating that implants should be contraindicated in these patients (2,13,14,16). Among the objectives of the protocols described is to inform the patient adequately of the risks that they take if they are wearers or dental implants are placed in relation to the antiresorptive treatment, carefully assessing the different risk factors involved, especially in patients with cancer.

In recent years, several cases have been published in which osteonecrosis occurred around implants that had been in place for several months or even years and correctly osseointegrated (13,14,16,28). It would be an implant presence-triggered osteonecrosis, compared to an implant surgery-triggered osteonecrosis. Escobedo *et al*. (17), in a literature review and own series, concluded that peri-implant ONJ occurred more frequently in cases where implants had been loaded at least one year earlier (74 cases compared to 27 cases related to implant insertion). Our protocols take on board this highly significant point, because implant-wearing patients who commence treatment with antiresorptives should be warned that the risk of suffering ONJ will always exist owing to the very presence of the implant, and not just by its placement, therefore one way of preventing it would be not to place implants in any patient who is to be treated or is being treated with these drugs. Similarly, it is essential to prevent any peri-implant infectious process in wearers, the risk being greater in patients with cancer.

The increase in risk of ONJ around already osseointegrated implants may be justified by bone remodelling being decreased, such that the peri-implant bone under constant demand from masticatory load could not respond properly to the functional needs and would necrotize. However, ONJ does not always occur, and furthermore different authors have shown an important reduction in the frequency of osteonecrosis in patients who undergo certain preventive measures to avoid infectious sites around their implants, which leads one to think that there must be something else facilitating the emergence of this condition around the implants. Indeed, several publications have suggested that peri-implantitis could be a risk factor for ONJ associated with implants, (9,14,16,28), which reinforces the importance that the infectious/inflammatory theory has been gaining in recent years to explain the etiopathogenesis of ONJ. Thus, Troeltzsch (31) studied a cohort of 316 patients diagnosed with ONJ, of whom 34 were dental implant wearers (117 implants). Of these, 56% (19 patients, 62 implants) developed ONJ around the implants, 56 of which had been placed before commencing antiresorptive treatment, the majority undergoing treatment for cancer, although three patients were being treated for osteoporosis. It should be emphasized that this author found that clinical and radiological signs of peri-implantitis were significantly associated with the emergence of peri-implant ONJ, that is, that said inflammatory process could be involved in the development of their osteonecrosis. The presence of an implant could represent a less resistant site for the development of ONJ, the bone being more vulnerable to infection due to remodelling being decreased, thus, peri-implantitis induced by bacterial plaque could trigger in this context a condition of osteonecrosis (1,14,15,18). Moreover, emphasis has been placed on the fact that the prejudicial effect of antiresorptives could be aggravated by acidic environments as a result of concomitant infectious processes –for example, peri-implantitis–, which makes the infectious/inflammatory process an important focus for understanding the etiopathogenesis of ONJ (9).

In terms of all the above, and considering the latest knowledge or scientific consensus on the role of bacterial plaque/biofilm in the etiopathogenesis of peri-implant disease, the possibility of preventing ONJ by controlling peri-implant disease would make a lot more sense so that proper control of peri-implant health using appropriate periodontal maintenance protocols could prevent the development of peri-implant mucositis or its transition to peri-implantitis per se, with the risk of it triggering a condition of ONJ in these patients. This is the reason for the inclusion of this type of measures in our protocols.

ONJ treatment using antiresorptives is a real challenge for professionals due to the large number of variables involved, the numerous therapeutic possibilities employed, and the enormous variability of the protocols used in the literature, with very disparate results. Furthermore, many of the treatments used, especially surgical ones’ entail in many cases a worsening of the condition with an extension of the lesion. In a study on different therapeutic management approaches in the literature, the authors grouped them into 7 different therapeutic protocols, of which the best results were obtained with conservative treatment, clinical and radiological follow-up, minimally invasive surgical treatment and coadjuvant measures (19). This shows the enormous variety of existing proposals and the difficulty in taking decisions when faced with a specific case of osteonecrosis. Furthermore, evidence has shown the efficacy of different preventive measures which have been applied to these patients achieving a decrease in the cases of ONJ, although, likewise on this topic there is enormous variability in studies and different protocols which make it hugely difficult to compare them and bring them together (38).

Different articles report the influence of preventive strategies in the reduction of the incidence of drug related ONJ. Thus, Ripamonti found an incidence of 7.8% of ONJ in patients with lung cancer which fell to 1.7% after the application of preventive measures (25). In patients with multiple myeloma, Dimopoulos obtained an incidence of ONJ of 26.3% in patients with no preventive strategies, which fell to 6.7% in those who did receive said measures (20). Mücke, in patients suffering bone metastases in prostate cancer, obtained an incidence of ONJ of 23.3% in patients who were reviewed once a year by their dentist, which fell to 2.2% in those for whom meticulous preventive follow-up at 3 months was undertaken (32). Authors such as Bramati (30) or Vandome (29) carried out work along the same lines obtaining similar results. Assessing the above articles overall, preventive measures achieved a reduction in the incidence of ONJ of 77.3%, compared to control groups (37). However, studies supporting this important reduction in the incidence of ONJ with the application of preventive measures, present many methodological discrepancies with each other, such that the type of measures applied and the means of executing them are different, therefore it is complicated to be able to compare the results or decide which of the published protocols is the most suiTable one to apply. This is the reason that made us consider trying to bring together all the published evidence, update it and draft global protocols, easy to implement in a dental clinic considering the different possibilities which may be presented with these patients.

It is important to highlight that the quality of evidence for most of the articles which apply preventive measures and which have been described in this study is poor, either because of the small sample size, the short follow-up times, the type of retrospective control used, the application of unclear follow-up protocols, etc., such that we coincide with the authors themselves when they point to the advisability of carrying out controlled randomized prospective studies with larger samples and longer follow-up times to be able to achieve high levels of evidence.

Several studies have shown the efficacy of antibiotic prophylaxis in those patients undergoing treatment with antiresorptives who need some oral surgery procedure. Normally these studies have been conducted in patients who have undergone dental extractions, achieving favourable results because no case of ONJ developed after the application of these protocols. Currently it is a widely accepted measure by the authors, not just for extractions but also when facing any oral surgery that these patients require. (2,25-27,32,33,36). For this reason, this measure has been included in our protocols for any oral surgery procedure, including extractions in these patients, depending on the different circumstances that may be presented arising from the type of treatment they received and the treatment stage at which they find themselves.

The application of these protocols requires an interdisciplinary team which can handle the various treatments and apply the measures contained in them. Thus, it will be necessary to have qualified hygienists who have an in-depth knowledge of the disease in order to provide proper reports to the patients and answer their queries. Similarly, they should have persuasive qualities and motivation to be able to make the patient aware of the importance of looking after their oral health and maintaining it in an optimal state. Likewise, generalists should be equally well trained for undertaking conservative treatments in the most non-traumatic way possible and be able to diagnose each lesion adequately in order to apply the most suiTable treatment that manages to avoid unnecessary extractions in the future. The team of surgeons will also have to be prepared to perform surgery with the same minimal trauma and with suiTable antibiotic prophylaxis. It is equally important to maintain contact with the medical team involved in the treatment of the underlying pathology, especially rheumatologists, oncologists, internists and gynaecologists. All the above requires a great staff learning and organization effort, continuous training and coordination of the whole team involved in the preventive management of these patients.
